# Evolution of Autonomous Selfing in Marginal Habitats: Spatiotemporal Variation in the Floral Traits of the Distylous *Primula wannanensis*

**DOI:** 10.3389/fpls.2021.781281

**Published:** 2021-12-16

**Authors:** Wei Zhang, Ying Feng Hu, Xiao He, Wei Zhou, Jian Wen Shao

**Affiliations:** ^1^College of Life Sciences, Anhui Normal University, Wuhu, China; ^2^Provincial Key Laboratory of Conservation and Utilization of Biological Resources, Wuhu, China; ^3^Germplasm Bank of Wild Species, Kunming Institute of Botany, Chinese Academy of Sciences, Kunming, China; ^4^CAS Key Laboratory for Plant Diversity and Biogeography of East Asia, Kunming Institute of Botany, Chinese Academy of Sciences, Kunming, China; ^5^Lijiang Forest Biodiversity National Observation and Research Station, Kunming Institute of Botany, Chinese Academy of Sciences, Lijiang, China

**Keywords:** delayed selfing, herkogamy, heterostyly, homostyly, reproductive assurance, pollinator limitation

## Abstract

Outcrossing plant species are more likely to exhibit autonomous selfing in marginal habitats to ensure reproduction under conditions of limited pollinator and/or mate availability. Distyly is a classical paradigm that promotes outcrossing; however, little is known about the variation in floral traits associated with distylous syndrome in marginal populations. In this study, we compared the variation in floral traits including stigma and anther height, corolla tube length, herkogamy, and corolla diameter between the central and peripheral populations of the distylous *Primula wannanensis*, and assessed the variation of floral traits at early and late florescence stages for each population. To evaluate the potential consequences of the variation in floral traits on the mating system, we investigated seed set in each population under both open-pollinated and pollinator-excluded conditions. The flower size of both short- and long-styled morphs was significantly reduced in late-opening flowers compared with early opening flowers in both central and peripheral populations. Sex-organ reciprocity was perfect in early opening flowers; however, it was largely weakened in the late-opening flowers of peripheral populations compared with central populations. Of these flowers, disproportionate change in stigma height (elongated in S-morph and shortened in L-morph) was the main cause of reduced herkogamy, and seed set was fairly high under pollinator-excluded condition. Our results provide empirical support for the hypothesis on the evolution of delayed autonomous selfing in marginal populations of distylous species. Unsatisfactory pollinator service is likely to have promoted reproductive assurance of distylous plants with largely reduced herkogamy mimicking “homostyles.”

## Introduction

Living organisms, especially plants, usually display a certain range of distribution (Sexton et al., 2009; Cross and Eckert, 2020), and their distribution patterns generally follow the “abundant center” model. According to this model, the population frequency, size, and density of a species are the highest at the center of its distribution range, which coincides with its geographical origin and the most favorable conditions, and decline toward the periphery (Haeck, 1982; Brown, 1984; Lawton, 1993; Sagarin and Gaines, 2002). Peripheral populations tend to be more rigorous, unpredictable, and consequently smaller and more temporally variable than central populations (Sagarin and Gaines, 2002; Orsenigo et al., 2014). Additionally, compared with central populations, peripheral populations are more prone to fix unique genotypes, morphologies, life histories, and biological tolerance (Grant and Antonovics, 1978; Blows and Hoffmann, 1993; Sagarin and Gaines, 2002; Sexton et al., 2009). These unique variations in marginal populations are associated with not only rapid natural adaptation and radical evolution but also speciation (Grundt et al., 2006; Eckert et al., 2008; Barrett and Harder, 2017). Therefore, comparing central and peripheral populations and understanding how organisms adapt to marginal habitats have been popular topics of research in the fields of ecology, evolutionary biology, and genetics (Eckert et al., 2008; Sexton et al., 2009; Villellas et al., 2013).

Mate and/or pollinator limitation is common in peripheral populations, resulting in reduced opportunities for outcrossing and selection of alleles that increase autonomous self-fertilization to provide reproductive assurance (Piper et al., 1986; Cruden and Lyon, 1989; Lloyd, 1992; Herlihy and Eckert, 2005; Eckert et al., 2006; Griffin and Willi, 2014). Nevertheless, selfing is often associated with negative fitness because of the accumulation of deleterious mutations and stochastic loss of beneficial mutations (Charlesworth and Charlesworth, 1987; Charlesworth and Willis, 2009; Wang et al., 2020). Moreover, selfing is reported to decrease the genetic variability and viability of populations in the long term (Takebayashi and Morrell, 2001; Goldberg et al., 2010; Igic and Busch, 2013). Autonomous selfing that occurs earlier or competes with outcrossing can decrease the offspring’s fitness because those self-fertilized ovules and pollen could otherwise be outcrossed (i.e., gamete discounting costs) (Lloyd, 1992; Eckert and Herlihy, 2004). However, after all outcrossing opportunities have been exhausted (i.e., delayed autonomous selfing), autonomous selfing that occurs at late florescence stages can provide the benefits of autogamy, avoiding discounting costs (Cruden and Lyon, 1989; Qu et al., 2007). Therefore, the relative timing of selfing events may be important for overall reproductive fitness, and delayed selfing seems ideally adaptive in habitats where pollination is scarce and/or unpredictable (Lloyd, 1992; Fenster and Martén-Rodríguez, 2007).

Heterostyly is a fascinating adaptation that promotes outbreeding, and a classical botanical paradigm that has arisen independently in at least 28 angiosperm families (Ganders, 1979; Lloyd and Webb, 1992a). Distyly, the most common form of heterostyly, is characterized by two different floral morphs within a population, i.e., long-styled (L) and short-styled (S) morphs, also known as pins and thrums, respectively (Darwin, 1877). Stigma is placed higher than the anthers in the L-morph, and lower than the anthers in the S-morph; this structural arrangement is called reciprocal herkogamy (Webb and Lloyd, 1986). Distyly is usually accompanied by diallelic self- and intra-morph incompatibility as well as ancillary floral polymorphisms, such as differences in pollen size, pollen number, and stigma shape (Dulberger, 1992). Reciprocal herkogamy is thought to be the key characteristic of the distylous syndrome and can promote cross-pollination and limit selfing and sexual interference (Darwin, 1877; Barrett and Shore, 2008; Keller et al., 2014). Therefore, it is generally assumed that the positions of stigmas and anthers are subject to strong selective pressures that maintain the floral polymorphism fixed and stable (Dulberger, 1992; Lloyd and Webb, 1992b). Empirical evidence suggests that substantial intraspecific floral variation occurred in some distylous species because of selection pressure under specific habitats, resulting in profound ecological and evolutionary significance (Richards and Koptur, 1993; Ferrero et al., 2009, 2011; Sampson and Krebs, 2012; Brys and Jacquemyn, 2015). However, to the best of our knowledge, the patterns of variation of morphological traits unique to the distylous floral syndrome have not yet been explicitly compared between peripheral and central populations, even though such comparisons have potential importance for explaining the ecological and evolutionary role of the heterostyly.

*Primula* (Primulaceae) is a typical distylous genus widely distributed in the temperate and arctic areas of the northern hemisphere, especially in the Himalayas and southwest China (Richards, 2003). Of the approximately 430 *Primula* species, approximately 385 are distylous, while 45 are homostylous, i.e., stigma and anthers are monomorphic and at the same height in the corolla tube (Mast et al., 2006; de Vos et al., 2014). High species diversity in the *Primula* provides ample opportunities for investigating the variation and maintenance mechanism of a distylous floral syndrome (Ganders, 1979; Richards, 2003). Many studies on the *Primula* have shown that a small number of floral traits, especially the relative position of reproductive organs, can remarkably affect the pollen transfer and mating patterns, thus having far-reaching ecological and evolutionary implications (e.g., Nishihiro et al., 2000; Keller et al., 2012, 2014; Brys and Jacquemyn, 2015; Liu et al., 2015; Deschepper et al., 2018; Jiang et al., 2018). Even in homostylous species, *Primula halleri*, small amounts of herkogamy variation during anthesis can have large effects on the reproductive strategy (de Vos et al., 2012, 2014). *Primula* serves as a model genus for studying the variation in the distylous floral syndrome and its ecological implications and has been the focus of attention since Darwin’s seminal work on heterostyly (Darwin, 1877; Mast et al., 2006; Gilmartin, 2015).

*Primula wannanensis* X. He and J. W. Shao, a newly described species based on morphological, molecular, and reproductive data (He et al., 2021), is restricted to hilly and mountainous regions in southern Anhui Province (eastern China), with Huangshan mountain located at the center of its geographical distribution (Shao et al., 2019). *Primula wannanensis* is an annual distylous herb that grows from August to June and exhibits only sexual reproduction *via* seeds (He et al., 2021). Compared with homomorphic species, distylous species are generally more sensitive to the scarcity of pollinators because of the spatial separation of sex organs. It is thus conceivable that the short-lived distylous herb, *P. wannanensis*, may strongly benefit from reproductive assurance in peripheral populations. In addition, as a typical insect-pollinated herb, *P. wannanensis* has a relatively long flowering period, with several umbellate inflorescences opening in succession (Hu and Kelso, 1996; Richards, 2003). Theoretical and empirical studies show that flowers opening at different time points are likely to show substantial variation in morphological traits because of variation in resource availability, plant condition, pollinator service, and mate availability among different seasons (Brunet and Charlesworth, 1995; Ishii and Sakai, 2002; Obeso, 2002; Diggle, 2003; Marshall et al., 2010). However, the variation of floral characters during the flowering period and whether these change affect sex organs spatial matching degree and their reproductive strategy in distyly remains unknown.

In this study, using *P. wannanensis* as the study system, we documented the variation in floral traits on a spatial scale (by comparing central and peripheral populations) as well as on a temporal scale (by comparing early and late florescence stages). To test the prediction that these floral traits variations tend to promote the occurrence of delayed autonomous selfing more easily in marginal populations than in central populations. Specifically, we address the following questions: (i) whether the distylous floral traits differ between marginal and central populations; (ii) whether the floral morphological traits differ between early and late-opening flowers, and if so, whether these temporal floral variation patterns differ spatially; and (iii) whether the floral morphological trait variation affects the seed set rate under open-pollinated and pollinator-excluded conditions. The results can shed some light on the adaptive strategies of the marginal populations and the maintenance or loss mechanism of the floral polymorphism in those typical outcrossing distylous plants (Barrett and Shore, 1987).

## Materials and Methods

### Study Material and Site Selection

*Primula wannanensis* usually grows on shaded slopes under or at the edge of deciduous, broad-leaved forests between 50 and 1,000 m a.s.l. (He et al., 2021). Plants mostly possess 10–30 pinnate compound leaves and 3–10 (up to 15) scapes, each bearing 6–9 flowers in 2–3 layers. The flowering period is long, with inflorescences blooming in succession from late February to mid-May. Flowers are pollinated primarily by the bee species, *Bombylius major* and *Anastoechus chinensis* (Shao et al., 2008). Controlled pollination indicates that the expression of self-incompatibility is not strict and varies extensively among individuals and populations; all distylous populations contain self- or partly self-compatible individuals, although their proportions are significantly higher in peripheral populations than in central populations (Shao et al., 2012, 2019). *Primula wannanensis* has two distinct populations, distylous and homostylous, based on the morph structure. Distylous populations contain almost equal proportions of L- and S-morphs, and are mainly distributed at (or near) the geographical distribution center (e.g., in and around the Huangshan mountain) and in marginal areas (e.g., near the Qidu township). Homostylous populations have monomorphic long or short styles (with stamens and stigma both at the mouth or middle of the corolla tube) and occur only in the most marginal areas of the distribution range (He et al., 2021). Four distylous populations of *P. wannanensis* were used in this study (Supplementary Table 1): two populations in the central distribution area (GCN and HDY), and two populations in the marginal distribution area (QDX and YLD). The GCN and HDY populations were found growing under temperate conditions in the mixed deciduous forest in the Huangshan mountain, whereas QDX and YLD populations grew along the roadside or near the edge of the forest on small hills near the Qidu township.

### Floral Trait Measurements

In mid-early March and mid-late April 2017, approximately 150 newly opened flowers were sampled from each population, with at least 0.5 m distance between two flowers. Because of differences in population area and plant density, the number of samples collected from each population varied from 61 to 365. In total, 1,242 flowers were sampled including 797 early opening flowers and 445 late-opening flowers. To minimize the influence of inflorescence structure on flower morphology, all flowers were picked from the first layer. Each flower was sliced open longitudinally and digitally photographed. The following six floral traits were measured or calculated: stigma height (SH), anther height (AH), corolla tube length (CTL), herkogamy (HE, i.e., stigma–anther separation), relative herkogamy (RHE; the rate of HE/CTL reflect whether the change in HE is proportional to the overall change in CTL), and corolla diameter (CD) (Supplementary Figure 1). All traits were measured to an accuracy of 0.01 mm using the ImageJ software (Rueden et al., 2017).

### Influence of Floral Trait Variation on Seed Set

Before the start of florescence (early March) in 2018, approximately 60 plants were marked in each population, and covered by a dense net (mesh diameter < 0.5 mm) to exclude pollinators (Shao et al., 2008). Based on the preliminary observation that the degree of herkogamy does not vary among flowers within a layer and does not change after flower opening (unpublished data), one flower from the first layer was picked from each marked plant at the early florescence stage in mid–early March for floral trait measurement, while another flower in the same layer was marked for testing the autonomous selfing capacity. One month later, late-opening flowers were observed and marked as described above. Floral traits were measured according to the above description. Fruits of marked flowers were collected in early and late May, respectively, and the number of seeds and undeveloped ovules per fruit was counted under a stereomicroscope (Shao et al., 2019). Additionally, to evaluate the seed setting ability of plants under natural conditions (with the participation of pollinators), approximately 60 plants were marked in each population, and their floral traits and seed setting ability were investigated in the same manner as that for plants covered with a net.

### Statistical Analyses

To obtain a general overview of the spatial and temporal variation in floral traits, principal component analysis (PCA) of the six floral traits was performed separately for each morph type at different sites and different flowering phases. To test whether the overall floral traits of distylous flowers differed between the central and marginal sites and between early and late flowering phases, multivariate analysis of variance (MANOVA) was performed, with the site, flowering phase, and site × flowering phase interaction as fixed factors. Subsequently, univariate analyses of variance were performed to identify the floral traits that differed significantly between the two sites and the two flowering phases. Tukey’s honestly significant difference (HSD) test for multiple comparisons was performed to determine the significance of differences in floral traits between the two sites in the same flowering phase or between the two flowering phases at the same site. Pearson correlation coefficient was calculated to determine the correlation among the floral traits of different morphs.

The indices developed by Sánchez et al. (2008) were used to compare the degree of spatial matching between the reciprocal arrangements of reproductive organs in different populations. These indices compare each organ position with every other organ position in the sample and provide a single combined metric of reciprocity (*R*) for the lower- and upper-level reproductive organs. These indices can be interpreted as a measure of the average population-level deviation from perfect reciprocity, with a value of 1 indicating perfect reciprocity, and values > 0.5 indicating distyly (Sánchez et al., 2008, 2013).

A generalized linear mixed (GLM) model, with Gaussian distribution and restricted maximum likelihood (REML), was used to test whether the seed set rate of plants covered with or without a net differed significantly between sites (central vs. marginal), flowering phases (early vs. late) and morphs (L- vs. S-morph). In this model, space, time, morph, and space × time were entered as fixed factors. Bonferroni correction was performed for multiple comparisons to determine the significant differences in seed set rate among different experimental treatments. A linear least regression model was used to test the relationship between herkogamy and the seed set rate, assuming a normal distribution of the errors. All statistical analyses were performed in SPSS version 19.0 (IBM Inc., Armonk, New York, United States).

## Results

### Spatial and Temporal Variation in Floral Traits

**FIGURE 1:**
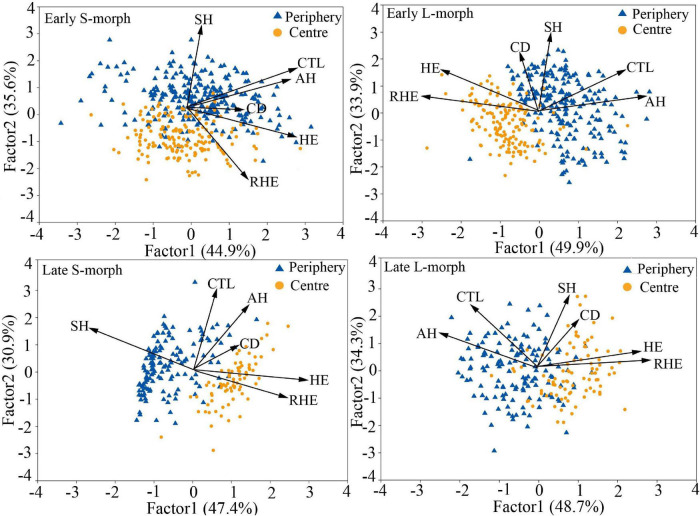
Principal component analysis (PCA) biplots of six floral traits of L-morph and S-morph individuals of *Primula wannanensis* in central and peripheral populations at different florescence stages. SH, stigma height; AH, anther height; CTL, corolla tube length; HE, herkogamy; RHE, relative herkogamy; CD, corolla diameter.

**FIGURE 2:**
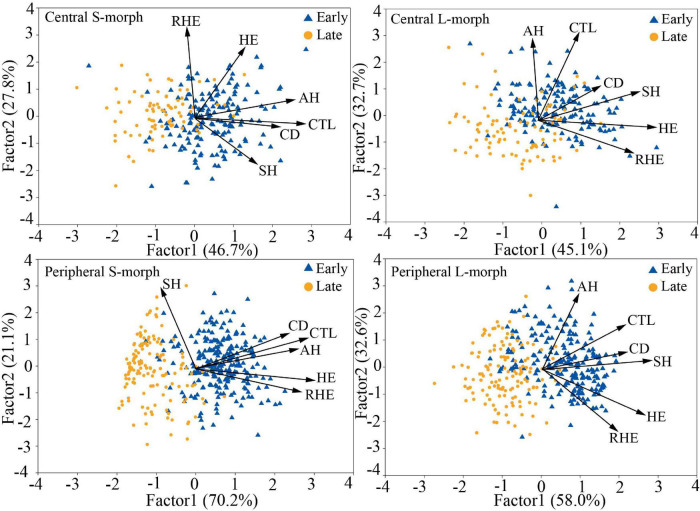
PCA biplots of six floral traits of L- and S-morphs of *P. wannanensis* at early and late florescence stages at different sites. SH, stigma height; AH, anther height; CTL, corolla tube length; HE, herkogamy; RHE, relative herkogamy; CD, corolla diameter.

**FIGURE 3:**
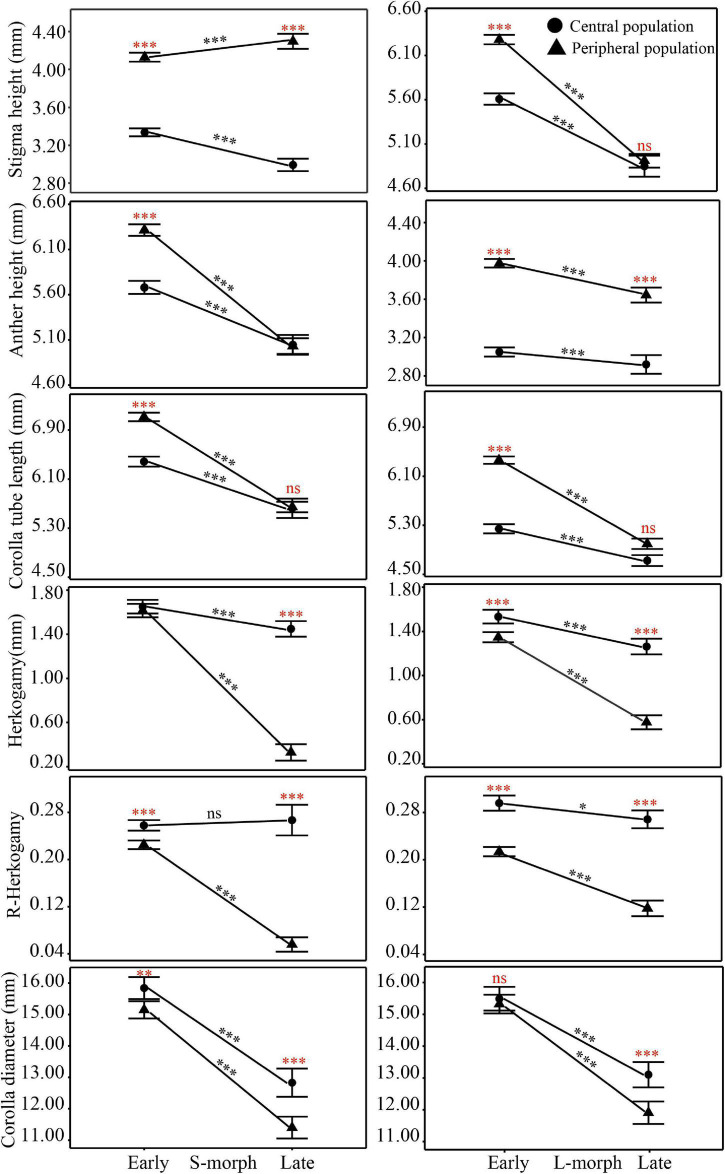
Spatiotemporal differences in floral traits of *P. wannanensis* plants between different sites and between different florescence stages. Data represent mean ± standard error (SE). The red asterisk (*) indicates significant differences between central and peripheral sites, and the black asterisk (*) indicates significant differences between early and late florescence phases. **indicate significance level: *P* < 0.01 and ***indicate significance level: *P* < 0.001.

The overall morphological traits of S- and L-morphs of *P. wannanensis* showed significant spatial variation [S-morph: Wilks’ λ = 0.32, *F*_(6, 630)_ = 227.86, *P* < 0.0001; L-morph: Wilks’ λ = 0.37, *F*_(6, 594)_ = 169.66, *P* < 0.0001] and temporal variation [S-morph: Wilks’ λ = 0.48, *F*_(6, 630)_ = 114.53, *P* < 0.0001; L-morph: Wilks’ λ = 0.44, *F*_(6, 594)_ = 125.35, *P* < 0.0001], along with significant space × time interaction (Table 1), indicating that site-specific differences in floral traits vary between different flowering phases. The results of PCA also showed obvious differences in floral traits of S- and L-morphs between the central and peripheral sites and between early and late flowering phases; the floral traits of the S-morph in marginal populations changed significantly during the late flowering phase (Figures 1, 2). In addition, the first and second principal components (PC1 and PC2, respectively) of all floral traits, except CD, contributed much to these spatiotemporal variations (Supplementary Tables 2, 3). Univariate tests indicated that all floral traits differed significantly between the central and peripheral sites (*P* < 0.05) and between early and late flowering phases (*P* < 0.05), and all traits, except CD of the S-morph, were significantly affected by the site × flowering phase interaction (Supplementary Table 4). Compared with central populations, both floral morphs in peripheral populations showed a significantly higher positioning of stigmas and anthers, with longer corolla tubes in the early opening flowers, but without larger herkogamy (conversely smaller in the L-morph), resulting in significantly smaller RHE value in peripheral populations (Figure 3). Compared with early opening flowers, the late-opening flowers showed significantly reduced CD, CTL, and SH in both morphs, and this declining trend was more striking in peripheral populations than in central populations (Figure 3). However, pistil showed significant differences between the two floral morphs; the SH of the L-morph decreased significantly, especially in marginal populations, whereas that of the S-morph decreased in central populations but increased significantly in marginal populations (Figure 3). Consequently, in central populations, the HE value of late-opening flowers decreased from 1.65 to 1.45 mm in the S-morph and from 1.53 to 1.27 mm in the L-morph, but the RHE value showed no significant decline in the S-morph (*P* > 0.05) and only a marginally significant decrease in the L-morph (*P* = 0.011). By contrast, in peripheral populations, the HE value of late-opening flowers decreased significantly from 1.35 to 0.58 mm in the L-morph and from 1.62 to 0.33 mm in the S-morph, resulting in a significant decline in the RHE value (Figure 3).

**TABLE 1:** Multivariate analysis of variance of the effects of the site (space), flowering phase (time), and their interaction on the overall floral traits of L- and S-morphs.

Morphs	Variable	Wilks’ λ	*d.f*	*F*	*P*
S-morph	Space	0.32	6,630	227.86	<0.0001
	Time	0.48	6,630	114.53	<0.0001
	Space * Time	0.71	6,630	43.32	<0.0001
L-morph	Space	0.37	6,594	169.67	<0.0001
	Time	0.44	6,594	125.35	<0.0001
	Space * Time	0.78	6,594	28.11	<0.0001

**FIGURE 4:**
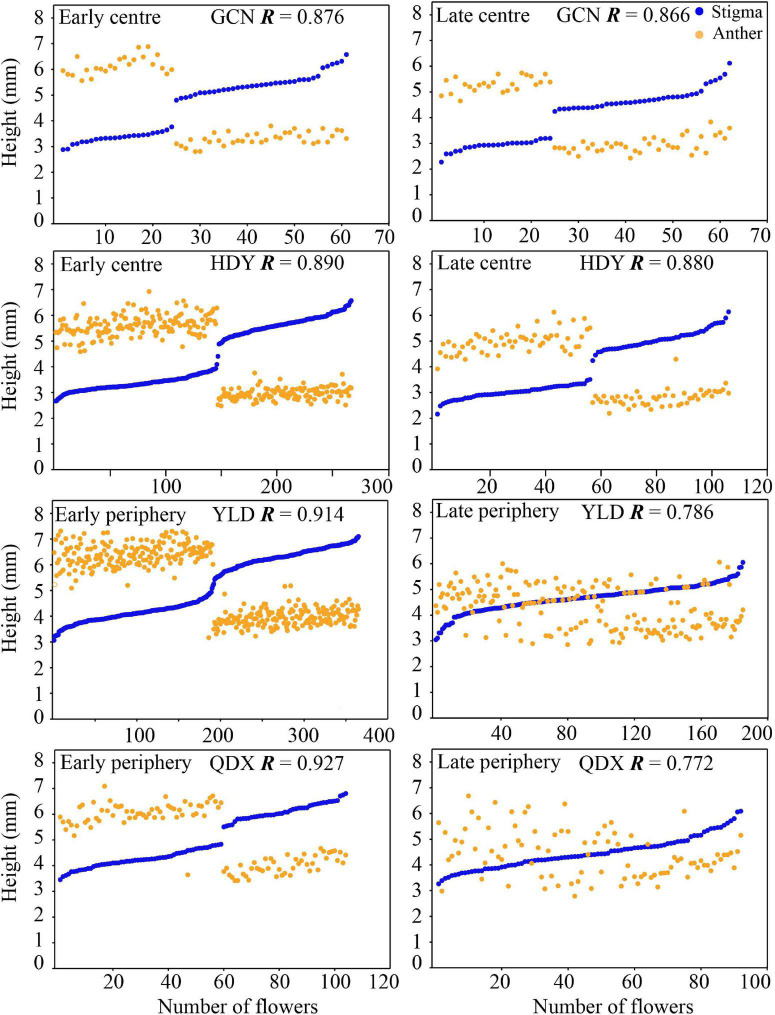
Scatterplots of relative stigma height (SH) and anther height (AH) of each sampled *P. wannanensis* plant at different flowering phases. *R* represents the global reciprocity index of Sánchez et al. (2013).

**FIGURE 5:**
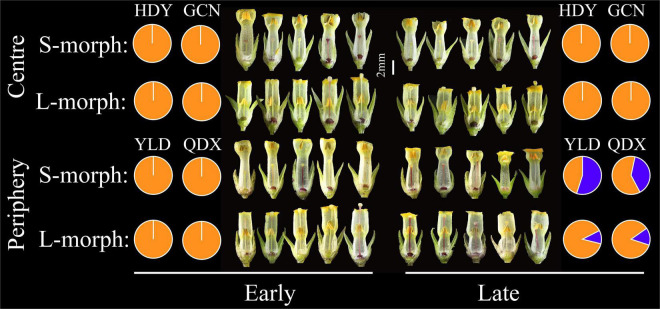
Spatiotemporal variation in floral traits of *P. wannanensis* plants. Orange and blue sections of the pie chart indicate herkogamy values greater than 0 mm and equal to 0 mm, respectively.

Floral trait variation during the early and late flowering phases in central populations showed discrete dimorphism, and all flowers showed a certain level of herkogamy, which changed from 0.57 to 3.15 mm (Figure 4). However, floral trait variation in late-opening flowers in peripheral populations appeared continuous, based on the obscure dimorphism variation pattern, and herkogamy disappeared in nearly one-third individuals (34.05% in YLD, and 26.09% in QDX; Figure 5). Consequently, the global indices of reciprocity (*R*), calculated according to Sánchez et al. (2013), were high (0.89–0.92) during the early and late flowering phases in central populations (HDY: 0.88, GCN: 0.87; Figure 4) but significantly lower during the late flowering phase in peripheral populations (YLD: 0.79, QDX: 0.77, *t*_6_ = 6.50, *P* < 0.001; Figure 4). The analysis of morphological data measured in 2018 (to investigate the influence of floral traits on seed setting ability) revealed that the spatiotemporal variation pattern in 2018 was consistent with that in 2017 (Supplementary Tables 6, 7 and Supplementary Figure 2), indicating that these spatial and temporal patterns of floral trait variation observed in *P. wannanensis* are stable.

### Influence of Floral Trait Variation on Seed Setting Ability

**FIGURE 6:**
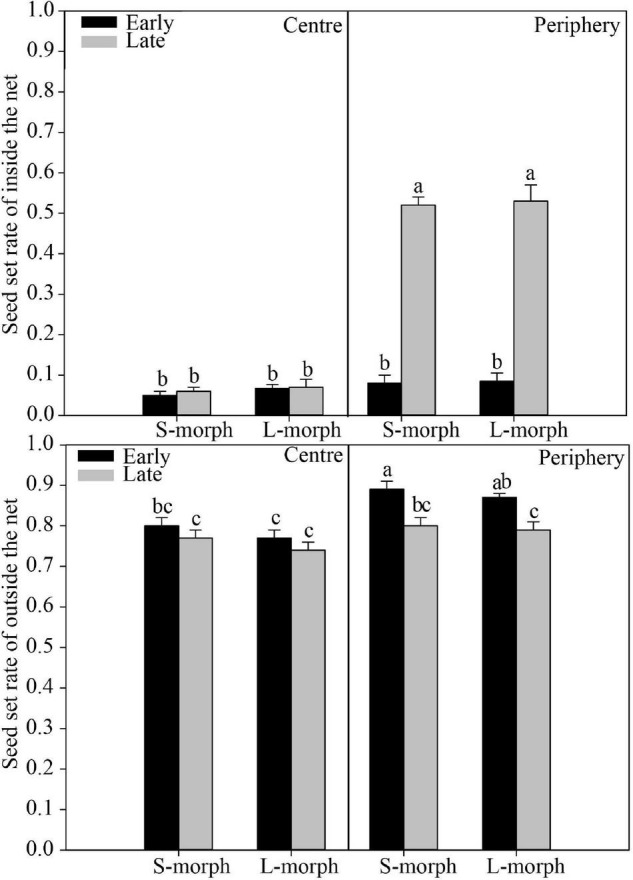
Seed set rate of the central and peripheral populations of *P. wannanensis* under different experimental treatments. Error bars indicate mean ± standard error (SE), and superscript lowercase letters indicate significant differences (*P* < 0.05).

Regardless of the presence or absence of effective pollinators, the seed set rate was significantly affected (*P* < 0.001) by space (central vs. peripheral site) and time (early vs. late flowering phase) but not by the morph (L- vs. S-morph) (Table 2). In addition, the interaction between space and time significantly (*P* < 0.001) affected the seed set rate only when plants were covered with a net to exclude pollinators (Table 2), indicating site-specific differences in seed set rate vary between different flowering phases in the absence of pollinators. When effective pollinators were excluded, the seed set rate in central populations was very low (average range: 0.05–0.08) and showed no significant differences between morphs (L- vs. S-morph) and between flowering phases (early vs. late) [*F*_(3, 274)_ = 0.41, *P* > 0.05; Figure 6]. The seed set rates of both morphs during the early flowering phase were also very low in peripheral populations (average range: 0.08–0.09), similar to those in central populations [*F*_(5, 381)_ = 0.60, *P* > 0.05]. However, the seed set rates of both morphs were quite high during the late flowering phase (average: 0.52 in the S-morph, 0.53 in the L-morph), which were significantly higher than those during the early flowering phase or those in central populations [S-morph: *F*_(1, 107)_ = 94.77, *P* < 0.001; L-morph: *F*_(1, 102)_ = 101.63, *P* < 0.001; Figure 6]. Under natural conditions, the seed set rates of both morphs in the central and peripheral populations were relatively high (means changed from 0.77 to 0.89; Figure 6). In central populations, the seed set rates of both morphs during the early flowering phase were similar to those during the late flowering phase [*F*_(3, 156)_ = 2.42, *P* > 0.05]. The seed set rates of both morphs during the early flowering phase in peripheral populations (S-morph: 0.89 and L-morph: 0.87) were significantly higher than those in central populations [S-morph: 0.80, *F*_(1, 78)_ = 15.37, *P* < 0.001; L: 0.77, *F*_(1, 78)_ = 23.72, *P* < 0.001] and those during the late flowering phase [S-morph: 0.80, *F*_(1, 78)_ = 12.07, *P* < 0.01; L-morph: 0.79, *F*_(1, 78)_ = 10.55, *P* < 0.01].

**TABLE 2:** Generalized linear mixed (GLM) model analysis of the effects of space (central vs. peripheral sites), time (early vs. late florescence) and morph (L- vs. S-morph) on seed set rate of *Primula wannanensis* plants covered with or without a net in natural populations.

Dependent variable	Source	*df*	Wald χ^2^	*P*
Seed set rate (inside the net)	Space	1	242.84	**<0.001**
	Time	1	218.56	**<0.001**
	Type	1	0.34	0.44
	Space * Time	1	204.23	**<0.001**
Seed set rate (outside the net)	Space	1	36.69	**<0.001**
	Time	1	26.13	**<0.001**
	Type	1	0.78	0.38
	Space * Time	1	2.49	0.11

*Values in bold indicate significant P-values (P < 0.05).*

**FIGURE 7:**
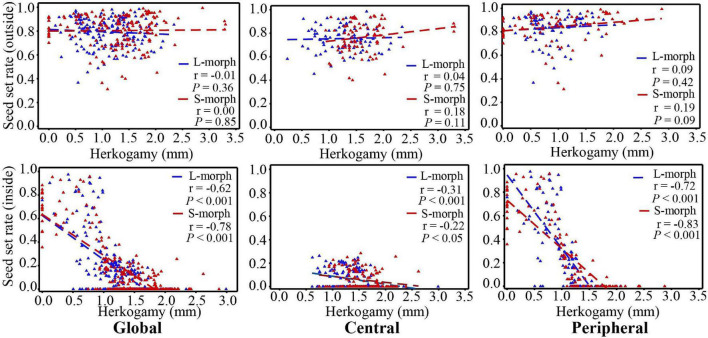
Correlation between seed setting rate and herkogamy of *P. wannanensis* plants covered or without a net. Dotted lines indicate linear regression.

**FIGURE 8:**
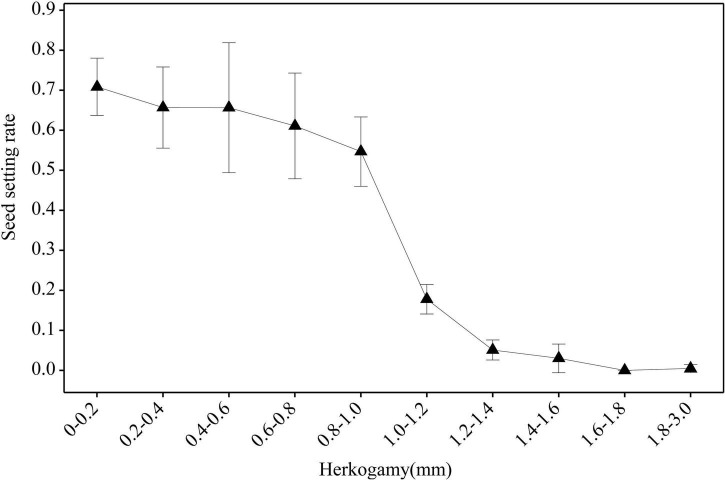
Effect of herkogamy on the seed setting rate of *P. wannanensis* plants covered with a net to exclude effective pollinators. The X-axis indicates the herkogamy value, and the Y-axis indicates the average seed setting rate. Error bars indicate a 95% confidence interval.

Under natural conditions, the seed set rates of both morphs showed no correlation with the herkogamy value (Figure 7). However, when effective pollinators were excluded with a net, the seed set rates of both morphs showed a significant negative correlation with the herkogamy value (L-morph: *r* = 0.62, *P* < 0.001; S-morph: *r* = 0.78, *P* < 0.001), and this negative correlation was stronger in peripheral populations (L-morph: *r* = 0.72, *P* < 0.001; S-morph: *r* = 0.83, *P* < 0.001) than that in central populations (L-morph: *r* = 0.31, *P* < 0.001; S-morph: *r* = 0.22, *P* < 0.05) (Figure 7). In peripheral populations, the average seed set rate was relatively high (range: 0.55–0.71; Figure 8) when the herkogamy value was less than 1.0 mm but decreased remarkably when the herkogamy value increased beyond 1.0 mm; average seed set rates of 0.18 and < 0.05 were observed at herkogamy values of 1.0–1.2 mm and > 1.2 mm, respectively (Figure 8).

## Discussion

Our experimental investigations of floral trait and mating system variation in the distylous species *P. wannanensis* revealed several novel findings. First, we showed that the size of late-opening flowers was significantly reduced in both marginal and central populations, and the HE value was more strongly decreased in peripheral populations than in central populations (Figure 3). Second, variation in stigma position was the main cause of reduction in HE, with the style greatly shortened in the L-morph but elongated in the S-morph flowers (Figures 3–5). Third, a high seed set through autonomous selfing was detected in the late-opening flowers of peripheral populations (Figures 6–8). Together, these results indicate that delayed autonomous selfing occurs in the marginal populations of *P. wannanensis* because of a reduction in HE in late-opening flowers. Below we compare the results of the current study with those of previous studies on floral trait and mating system variation in distylous plant species and discuss their ecological and evolutionary implications.

### Spatial Variation in Floral Traits at the Early Flowering Stage

In general, relatively sufficient pollinators and resources required for seed set are available at the peak or earlier flowering time, which is very important for most plants to execute sexual reproduction (Elzinga et al., 2007; Marshall et al., 2010). In our study system, variation in the position of sexual organs in both morphs in central and peripheral populations showed obvious discrete dimorphism (Figure 4) at the early flowering stage, with high global *R* indices (>0.87), indicating a good match between male and female organs. Furthermore, the seed set rate less than 10% of these flowers when effective pollinators are excluded with a net (Figure 6), indicating they were lack of effective automatic pollination mechanism and their effective pollen transfer normally requires pollinators activity. Consequently, regardless of the site, the early flowers of *P. wannanensis*, similar to those of most other distylous species, showed typical reciprocal herkogamy characteristics that promote disassortative pollination and reduce sexual interference (Ganders, 1979; Keller et al., 2014; Zhou et al., 2015; Barrett, 2019).

Highly significant correlations were detected between CTL and the positions of sexual organs in both morphs (Supplementary Table 5), as documented in other distylous *Primula* species, suggesting that corolla tube elongation plays an important role in the spatial arrangement of sexual organs in the *Primula* genus (Kálmán et al., 2007; Zhu et al., 2009; Keller et al., 2012; Brys and Jacquemyn, 2015). Interestingly, compared with central populations, marginal populations exhibited longer corolla tubes and higher positioning of stigmas and anthers in both morphs (Figure 3). Preliminary transplanting experiments in the greenhouse showed that this site-specific variation was stable under the same cultivation conditions (Zhang and Shao, unpublished data), indicating that these differences were a result of long-term local adaptive selection and represent heritable variation. Floral traits are largely attributed to phenotypic selection in plant–pollinator interactions (Boberg et al., 2014; Huang et al., 2016; Wu and Li, 2017). Previous studies showed that the two long-tongued bee species, *B. major* and *A. chinensis*, are effective pollinators of *P. wannanensis*, and the proboscis of the former bee species (average length: ∼5.78 mm) is significantly shorter than that of the latter (average length: ∼7.90 mm) (Shao et al., 2008). In the current study, we could not determine whether pollinator communities varied between central and peripheral populations. Further investigation is needed to determine whether the observed site-specific variation could be attributed to interactions with local pollinators, although coevolutionary between some key floral traits (such as corolla tube or stigma position) and local pollinators has been documented (Moeller, 2006; Paudel et al., 2015, 2016), even including some primroses (Brys and Jacquemyn, 2015; Wu and Li, 2017). It should be noted that the relatively narrow corolla tube, as in *P. wannanensis* (<2 mm diameter), may restrict the entry/exit path of the proboscis of effective pollinators foraging for nectar secreted at the bottom of the corolla. Higher position of sex organs in a floral tube may lead to a larger area of contact with the proboscis of pollinators, thus increasing pollination efficiency (Nishihiro et al., 2000). Thus, in peripheral populations, where pollinators were usually limited and unpredictable, higher CTL associated with higher stigma and anther positions may be selected to improve the efficiency of pollination, which may explain why the seed set rate of early flowers under natural conditions was slightly higher in peripheral populations than in central populations in 2018 (Figure 6).

### Temporal Variation in Floral Traits

Although most *Primula* species have several inflorescences opening in succession, and thus have a relatively long flowering period (Hu and Kelso, 1996; Richards, 2003), floral trait variation in these distylous species during the flowering period and its reproductive implications have not yet been reported. Here, we documented a strong temporal difference in flower morphology in *P. wannanensis*. Compared with early opening flowers, the late-opening flowers showed significantly smaller reproductive organs (Figure 3), consistent with the major trend variation revealed in other sequentially blooming plants (Guitián and Navarro, 1996; Diggle, 2003; Bateman and Rudall, 2006). Furthermore, this temporal variation pattern of flowers showed conspicuous spatial differences. In the central populations, the measured floral traits of distylous such as (SH,AH, CTL, HE, and CD) all most scaled down in late opening flowers, thus causing the herkogamy near proportionally declined with floral tube (Figure 3). However, in peripheral populations, the SH variable did not scale down with other floral traits (AH, CTL, and CD), but the style was elongated in the S-morph and shortened in the L-morph (Figure 3), resulting in significantly decreased values of both HE and RHE (Figure 3). In addition, at the early flowering stage, the flowers outside the net could form seeds, while those covered with a net hardly formed seeds because of the exclusion of effective pollinators. However, at the late flowering stage, we did not document that the size of the flowers inside the net was significantly larger (*P* > 0.05) than that outside the net. Thus, whether the early opening flowers bear seeds or not had little effect on the size of late-opening flowers, suggesting that architectural effects, rather than phenotypic plasticity, could explain the decline in the size of late-opening flowers (Stephenson, 1981; Diggle, 1995, 2003).

The site-specific temporal variation in floral traits also led to a remarkable variation in the reproductive strategy of late-opening flowers. In central populations, the late-opening flowers maintained typical distylous floral characteristics (with relatively high reciprocal herkogamy, *R* > 0.86; Figure 4), and could not bear seeds when pollinators were excluded (Figure 6), implying that the floral traits of late-opening flowers promoted outcrossing. By contrast, in peripheral populations, the late-opening flowers did not show typical distylous floral traits, and could produce seeds by automatic self-pollination when pollinators were excluded because of the strong reduction or disappearance of herkogamy (Figure 6). Notably, the arrangement of sexual organs in the most late-opening flowers in marginal populations has changed to favor automatic selfing. The occurrence of self-pollination in only peripheral populations is consistent with the prediction that peripheral populations are more prone to suffer from pollinator scarcity or conspecific plants, and reproductive assurance is more likely to play a role at the ecological edge of the species distribution range (Brown, 1984; Busch, 2005; Griffin and Willi, 2014). Among a variety of self-pollination modes, delayed autonomous selfing is generally regarded as an adaptive strategy, as it provides reproductive assurance without incurring pollen and/or seed discounting costs (Schoen and Brown, 1991; Lloyd, 1992). Therefore, many studies report that species possess herkogamous flowers with sundry mechanisms to bring the reproductive organs together usually toward the end of the floral life cycle, e.g., by corolla dragging, which involves movement of the stigma toward the anthers or vice versa (Fenster and Martén-Rodríguez, 2007). In our current study, late-opening flowers were facilitated to undergo autonomous self-pollination, which differs from the delayed self-pollination that generally occurs at the end of the flower life cycle, the ecological significance of both mechanisms is the same. For *P. wannanensis*, the seed set rate of open-pollinated flowers (especially early opening flowers) in peripheral populations was high (Figure 6) and didn’t lower than that in central populations, raising doubts about the supposed limited pollinator service in peripheral populations (at least in 2018). Previous manual pollination experiments also revealed that outcrossing pollen grains possess a higher germination rate and pollen tube growth than that of selfing (Shao et al., 2011). Moreover, peripheral populations still contained relatively high genetic diversity and normal flower morph ratio (Shao et al., 2008, 2019). All these indicated that most of the seeds set by late flowers in peripheral populations might well result from outcrossing under natural conditions, although the actual contribution to autonomous pollination to seed set in late flowers in peripheral populations will require further molecular analysis of the offspring. Thus the autonomous selfing of late-opening flowers in peripheral populations serves as only a potential (or standby) reproductive assurance mechanism. Only when pollinators are scarce throughout the flowering period, the seeds produced by this self-pollinating system can be promoted, which provides a reproductive assurance and maintains population stability. Thus, the autonomous self-pollination capacity of late-opening flowers in marginal populations of *P. wannanensis* is another delayed reproductive assurance mode.

### Effects of Herkogamy on the Mating System in Distylous Species

In typical distylous species, mating patterns are rarely affected by herkogamy variation because of their strict sporophytic self-incompatibility, although small changes in reciprocal herkogamy significantly influence the efficiency of legitimate pollen deposition on stigmas (Ganders, 1979; Brys and Jacquemyn, 2015; Liu et al., 2015). According to our current observations, we conform this prediction, i.e., the selfing seed set rate is low and effected by herkogamy variation is very limited in central populations (Figure 5), which possess a relatively strict self-incompatibility system (Shao et al., 2019). However, it is largely unknown whether and to what extent the variation in herkogamy affects opportunities for autonomous selfing and reproductive assurance in heterostylous plants with self- or partially self-compatible systems, although the self-incompatibility of quite a few distylous species is not strictly (Riveros et al., 1995), even in the typical distylous genus *Primula* (Wedderburn and Richards, 1990; Barrett and Cruzan, 1994; Riveros et al., 1995; Yuan et al., 2017; Zhou et al., 2017; Jiang et al., 2018; Shao et al., 2019). In the marginal populations of *P. wannanensis*, self-incompatibility is fairly weak (Shao et al., 2019), and we found that the degree of herkogamy directly affected the autonomous selfing capacity of plants in the absence of pollinators (Figure 7). Further analysis revealed that the average seed set rate *via* selfing was relatively high (> 0.55 for all herkogamy classes; Figure 8) and showed no significant difference among herkogamy classes with separation less than 1.0 mm; however, the seed set rate sharply decreased to less than 0.10 with an increase in separation (Figure 8). This sharp decline in the seed set rate could be explained based on the assumption that the HE value of 1.0 mm possibly represents an approximate threshold, consistent with previous studies on the homostylous species *P. halleri* (de Vos et al., 2014, 2018) and distylous species *Primula chungensis* (Jiang et al., 2018), suggesting that this threshold likely has wider applicability, although experimental confirmation is needed.

### Evolution of Selfing in Distylous Species

Autonomous selfing is a common strategy employed by plants to accommodate an unpredictable pollinator environment because selfed seeds can provide reproductive assurance (Lloyd, 1992; Barrett, 2002). The spatial and/or temporal separation of female and male organs within flowers of self-compatible plants is generally assumed to have a direct impact on the degree of selfing and the capacity to autonomous self-pollination (Barrett, 2002; Nishihiro et al., 2000; Brys and Jacquemyn, 2011; de Vos et al., 2014, 2018). In distylous species, a commonly exhibited heteromorphic incompatibility system prevents self- and intramorph mating, and a significant component of phenotypic disassortative mating is maintained under the interaction of pollinator and reciprocal herkogamy in distylous species exhibiting self- and/or intramorph compatibility (e.g., *Luculia pinceana*; Zhou et al., 2015). Most heterostylous groups contain species that are monomorphic for style length, with anthers and stigmas positioned close together within a flower. These plants are generally self-compatible and predominantly exhibit selfing as a result of autonomous self-pollination (Ganders, 1979). As the main form of selfing variants, homostylous species are evolutionarily derived from distylous ancestors. In *Primula*, phylogenetic analyses demonstrated a single origin of distyly but numerous independent transitions to homostyly (Mast et al., 2006; de Vos et al., 2014; Zhong et al., 2019). Investigations of the genetic basis of homostyly in *Primula* indicated that the rapid transition from outcrossing to selfing was caused by loss-function mutations in a single-gene (*CYP734A50*), which controls the stigma height in the S-morph (Huu et al., 2016; Kappel et al., 2017). Consistent with the theoretical expectations of the genomic selfing syndrome (Cutter, 2019), a recent analysis of genome-wide molecular evolution revealed the maladaptation of autonomous selfing in homostylous species with strongly reduced genetic diversity, decreased purifying selection efficacy, and low adaptive evolution rates (Wang et al., 2020).

Delayed autonomous selfing has not been previously reported in distylous taxa, although studies have suggested different levels of autonomous selfing in derived lineages of homostylous species including *Primula* (Yuan et al., 2017; Zhong et al., 2019). Here, we report a novel example of delayed autonomous selfing occurring in the marginal populations of distylous *P. wannanensis*. Previously, Shao et al. (2019) reported that self-incompatibility was largely weakened in the marginal habitat of *P. wannanensis*; however, it is unclear what mode of selfing has been adopted to cope with the unstable pollination environment, i.e., pollinator facilitated selfing/geitonogamy, prior autogamy, simultaneous autogamy or delayed autogamy. All these options may provide the advantage of reproductive assurance, as long as the fitness of selfed progeny (ω_s_) to that of outcrossed progeny (ω_o_) exceeds 0.5 (ω_s_/ω_*o*_ > 0.5), i.e., inbreeding depression (δ = 1–ω_s_/ω_o_) is less than 0.5. In this study, late-opening flowers in marginal populations showed a high seed set rate under pollinator-excluded conditions (Figure 6); however, the early opening flowers maintained a high level of reciprocal herkogamy and consequently pollinator-mediated disassortative mating (i.e., outcrossing) (Figure 4). This implies a transition of mating strategy from obligate outcrossing in central populations to mixed-mating in marginal habitats, further suggesting that the selfing component of mixed-mating is more likely to occur in late-opening flowers, which is thus referred to as delayed autonomous selfing. This transition was distinctly different from the most common events of the mating system shift from outcrossing to selfing associated with the breakdown of distyly to homostyly. Predominant self-fertilization of homostyly may result in the accumulation of genetic load, leading to a “dead-end” over the long run (Wang et al., 2020). However, the ability to exhibit delayed autonomous selfing may represent a floral strategy that limits the most harmful genetic consequences of selfing and increases the long-term adaptive potential of the species.

## Data Availability Statement

The original contributions presented in the study are included in the article/Supplementary Material, further inquiries can be directed to the corresponding author/s.

## Author Contributions

JS conceived and designed the research. WZha, XH, and YH performed field experiments. WZha conducted data analyses and drafted the manuscript. JS and WZha (Kunming Institute of Botany) revised the manuscript. All authors contributed to the article and approved the submitted version.

## Conflict of Interest

The authors declare that the research was conducted in the absence of any commercial or financial relationships that could be construed as a potential conflict of interest.

## Publisher’s Note

All claims expressed in this article are solely those of the authors and do not necessarily represent those of their affiliated organizations, or those of the publisher, the editors and the reviewers. Any product that may be evaluated in this article, or claim that may be made by its manufacturer, is not guaranteed or endorsed by the publisher.

## References

[B1] Barrett S. C. H. (2002). The evolution of plant sexual diversity. *Nat. Rev. Genet.* 3 274–284. 10.1038/nrg776 11967552

[B2] Barrett S. C. H. (2019). ‘A most complex marriage arrangement’: recent advances on heterostyly and unresolved questions. *New Phytol.* 224 1051–1067. 10.1111/nph.16026 31631362

[B3] Barrett S. C. H. Cruzan M. (1994). “Incompatibility in heterostylous plants,” in *Genetic Control of Self-incompatibility and Reproductive Development in Flowering Plants*, eds Williams E. G. Clarke A. E. Knox R. B. (Dordecht: Springer Netherlands), 189–219.

[B4] Barrett S. C. H. Harder L. D. (2017). The ecology of plant mating and its evolutionary consequences in seed plants. *Annu. Rev. Ecol. Syst.* 48 135–157. 10.1146/annurev-ecolsys-110316-023021

[B5] Barrett S. C. H. Shore J. S. (1987). Variation and evolution of breeding systems in the *Turnera ulmifolia* L. Complex (Turneraceae). *Evolution* 41 340–354. 10.1111/j.15585646.1987.tb05802.x 28568757

[B6] Barrett S. C. H. Shore J. S. (2008). “New insights on heterostyly: comparative biology, ecology and genetics,” in *Self-incompatibility in Flowering Plants: Evolution, Diversity and Mechanisms*, ed. Franklin-Tong V. E. (Berlin: Springer-Verlag), 3–32.

[B7] Bateman R. M. Rudall P. J. (2006). Evolutionary and morphometric implications of morphological variation among flowers within an inflorescence: a case-study using European orchids. *Ann. Bot.* 98 975–993. 10.1093/aob/mcl191 17018569 PMC2803595

[B8] Blows M. W. Hoffmann A. A. (1993). The genetics of central and marginal populations of *Drosophila serrata*. I. Genetic variation for stress resistance and species borders. *Evolution* 47 1255–1270. 10.2307/2409990 28564275

[B9] Boberg E. Alexandersson R. Jonsson M. Maad J. Ågren J. Nilsson L. A. (2014). Pollinator shifts and the evolution of spur length in the moth-pollinated orchid *Platanthera bifolia*. *Ann. Bot.* 2 267–275. 10.1093/aob/mct217 24169591 PMC3890388

[B10] Brown J. (1984). On the relationship between abundance and distribution of species. *Am. Nat.* 124 255–279. 10.1086/284267

[B11] Brunet J. Charlesworth D. (1995). Floral sex allocation in sequentially blooming plants. *Evolution* 49 70–79. 10.1111/j.1558-5646.1995.tb05959.x 28593669

[B12] Brys R. Jacquemyn H. (2011). Variation in the functioning of autonomous self-pollination, pollinator services and floral traits in three *Centaurium* species. *Ann. Bot.* 107 917–925. 10.1093/aob/mcr032 21320880 PMC3080621

[B13] Brys R. Jacquemyn H. (2015). Disruption of the distylous syndrome in *Primula veris*. *Ann. Bot.* 115 27–39. 10.1093/aob/mcu211 25429005 PMC4284109

[B14] Busch J. W. (2005). The evolution of self-compatibility in geographically peripheral populations of *Leavenworthia alabamica* (Brassicaceae). *Am. J. Bot.* 92 1503–1512. 10.3732/ajb.92.9.1503 21646168

[B15] Charlesworth D. Charlesworth B. (1987). Inbreeding depression and its evolutionary consequences. *Annu. Rev. Ecol. Syst.* 18 237–268. 10.1146/annurev.es.18.110187.001321

[B16] Charlesworth D. Willis J. (2009). The genetics of inbreeding depression. *Nat. Rev. Genet.* 10 783–796. 10.1038/nrg2664 19834483

[B17] Cross R. L. Eckert C. G. (2020). Integrated empirical approaches to better understand species’ range limits. *Am. J. Bot.* 107 12–16. 10.1002/ajb2.1400 31828769

[B18] Cruden R. W. Lyon D. L. (1989). “Facultative xenogamy: examination of a mixed mating system,” in *The Evolutionary Ecology of Plants*, eds Bock J. H. Linhart Y. B. (Boulder, CO: Westview Press), 171–207.

[B19] Cutter A. D. (2019). Reproductive transitions in plants and animals: selfing syndrome, sexual selection, and speciation. *New Phytol.* 224 1080–1094. 10.1111/nph.16075 31336389

[B20] Darwin C. (1877). *The Different Forms of Flowers on Plants of the Same Species.* London: John Murray.

[B21] Deschepper P. Jacquemyn H. Brys R. (2018). The impact of flower morphology and pollinator community composition on pollen transfer in the distylous *Primula veris*. *Bot. J. Linn. Soc.* 186 414–424. 10.1093/botlinnean/box097

[B22] de Vos J. M. Ketter B. Isham S. T. Kelso S. Conti E. (2012). Reproductive implications of herkogamy in homostylous primroses: variation during anthesis and reproductive assurance in alpine environments. *Funct. Ecol.* 26 854–865. 10.1111/j.1365-2435.2012.02016.x

[B23] de Vos J. M. Keller B. Zhang L. R. Nowak M. D. Conti E. (2018). Mixed mating in homostylous species: genetic and experimental evidence from an alpine plant with variable herkogamy, *Primula halleri*. *Int. J. Plant Sci.* 179 87–99. 10.1086/695527

[B24] de Vos J. M. Wuest R. O. Conti E. (2014). Small and ugly? Phylogenetic analyses of the “selfing syndrome” reveal complex evolutionary fates of monomorphic primrose flowers. *Evolution* 68 1042–1057. 10.1111/evo.12331 24325205

[B25] Diggle P. K. (1995). Architectural effects and the interpretation of patterns of fruit and seed development. *Annu. Rev. Ecol. Syst.* 26 531–552. 10.1146/annurev.es.26.110195.002531

[B26] Diggle P. K. (2003). “Architectural effects on floral form and function: a review,” in *Deep Morphology: Toward a Renaissance of Morphology in Plant Systematics*, eds Stuessy T. F. Hörandl E. Mayer V. (Koenigstein: Gantner Verlag), 63–80.

[B27] Dulberger R. (1992). “Floral polymorphisms and their functional significance in the heterostylous syndrome,” in *Evolution and Function of Heterostyly*, ed. Barrett S. C. H. (Berlin: Springer-Verlag), 41–84.

[B28] Eckert C. C. Herlihy C. R. (2004). Using a cost-benefit approach to understand the evolution of self-fertilization in plants: the perplexing case of *Aquilegia canadensis* (Ranunculaceae). *Plant Species Biol.* 19 159–173. 10.1111/j.1442-1984.2004.00112.x

[B29] Eckert C. G. Samis K. E. Dart S. (2006). “Reproductive assurance and the evolution of uniparental reproduction in flowering plants,” in *Ecology and Evolution of Flower*, eds Harder L. D. Barrett S. C. H. (New York, NY: Oxford University Press), 183–203.

[B30] Eckert C. G. Samis K. E. Lougheed S. C. (2008). Genetic variation across species’ geographical ranges: the central–marginal hypothesis and beyond. *Mol. Ecol.* 17 1170–1188. 10.1111/j.1365-294X.2007.03659.x 18302683

[B31] Elzinga J. A. Atlan A. Biere A. Gigord L. Weis A. E. Bernasconi G. (2007). Time after time: flowering phenology and biotic interactions. *Trends Ecol. Evol.* 22 432–439. 10.1016/j.tree.2007.05.006 17573151

[B32] Fenster C. B. Martén-Rodríguez S. (2007). Reproductive assurance and the evolution of pollination specialization. *Int. J. Plant Sci.* 2 215–228. 10.1086/509647

[B33] Ferrero V. Chapela I. Arroyo J. Navarro L. (2011). Reciprocal style polymorphisms are not easily categorised: the case of heterostyly in *Lithodora* and *Glandora* (Boraginaceae). *Plant Biol.* 13 7–18. 10.1111/j.1438-8677.2009.00307.x 21134082

[B34] Ferrero V. de Vega C. Stafford G. I. Van Staden J. Johnson S. D. (2009). Heterostyly and pollinators in *Plumbago auriculata* (Plumbaginaceae). *S. Afr. J. Bot.* 75 778–784. 10.1016/j.sajb.2009.06.014

[B35] Ganders F. R. (1979). The biology of heterostyly. *N. Z. J. Bot.* 17 607–635. 10.1080/0028825X.1979.10432574

[B36] Gilmartin P. M. (2015). On the origins of observations of heterostyly in *Primula*. *New Phytol.* 208 39–51. 10.1111/nph.13558 26255981

[B37] Goldberg E. E. Kohn J. R. Lande R. Robertson K. A. Smith S. A. (2010). Species selection maintains self-incompatibility. *Science* 330 493–495. 10.1126/science.1194513 20966249

[B38] Grant M. C. Antonovics J. (1978). Biology of ecologically marginal populations of *Anthoxanthum odoratum*. I. Phenetics and dynamics. *Evolution* 32 822–838. 10.1111/j.1558-5646.1978.tb04637.x 28567929

[B39] Griffin P. C. Willi Y. (2014). Evolutionary shifts to self-fertilisation restricted to geographic range margins in North American *Arabidopsis lyrata*. *Ecol. Lett.* 17 484–490. 10.1111/ele.12248 24428521

[B40] Grundt H. H. Kjolner S. Borgen L. (2006). High biological species diversity in the Arctic flora. *Proc. Natl. Acad. Sci. U.S.A.* 103 972–975. 10.1073/pnas.0510270103 16418291 PMC1348009

[B41] Guitián J. Navarro L. (1996). Allocation of reproductive resources within inflorescences of *Petrocoptis grandiflora* (Caryophyllaceae). *Can. J. Bot.* 74 1482–1486. 10.1139/b96-178

[B42] Haeck H. J. (1982). The distribution of abundance. I. Measurements. *J. Biogeogr.* 9 303–316. 10.2307/2844717

[B43] He X. Cao J. J. Zhang W. Li Y. Q. Zhang C. Li X. H. (2021). Integrative taxonomy of herbaceous plants with narrow fragmented distributions: a case study on *Primula merrilliana* species complex. *J. Syst. Evol.* 10.1111/jse.12726

[B44] Herlihy C. R. Eckert C. G. (2005). Evolution of self-fertilization at geographical range margins? A comparison of demographic, floral, and mating system variables in central vs. peripheral populations of *Aquilegia canadensis* (Ranunculaceae). *Am. J. Bot.* 92 744–751. 10.3732/ajb.92.4.744 21652454

[B45] Hu C. M. Kelso S. (1996). “Primulaceae,” in *Flora of China*, eds Wu Z. Y. Raven P. H. Hong D. Y. (Beijing: Science Press), 39–78.

[B46] Huang S. Q. Wang X. P. Sun S. G. (2016). Are long corolla tubes in *Pedicularis* driven by pollinator selection? *J. Integr. Plant Biol.* 58 698–700. 10.1111/jipb.12460 26714618

[B47] Huu C. Kappel C. Keller B. Sicard A. Takebayashi Y. Breuninger H. (2016). Presence versus absence of CYP734A50 underlies the style-length dimorphism in primroses. *eLife* 5:e17956. 10.7554/eLife.17956 27596932 PMC5012859

[B48] Igic B. Busch J. W. (2013). Is self-fertilization an evolutionary dead end? *New Phytol.* 198 386–397. 10.1111/nph.12182 23421594

[B49] Ishii H. S. Sakai S. (2002). Temporal variation in floral display size and individual floral sex allocation in racemes of *Narthecium asiaticum* (Liliaceae). *Am. J. Bot.* 89 441–446. 10.3732/ajb.89.3.441 21665640

[B50] Jiang X. F. Zhu X. F. Li Q. J. (2018). Variation in the degree of reciprocal herkogamy affects the degree of legitimate pollination in a distylous species. *AoB Plants* 10:ly022. 10.1093/aobpla/ply022 29765587 PMC5941151

[B51] Kálmán K. Medvegy A. Pénzes Z. Mihalik E. (2007). Morph-specific variation of floral traits associated with reciprocal herkogamy in natural populations of *Primula vulgaris* and *Primula veris*. *Plant Syst. Evol.* 268 15–27. 10.1007/s00606-007-0575-5

[B52] Kappel C. Huu C. N. Lenhard M. (2017). A short story gets longer: recent insights into the molecular basis of heterostyly. *J. Exp. Bot.* 68 5719–5730. 10.1093/jxb/erx387 29099983

[B53] Keller B. de Vos J. M. Conti E. (2012). Decrease of sexual organ reciprocity between heterostylous primrose species, with possible functional and evolutionary implications. *Ann. Bot.* 110 1233–1244. 10.1093/aob/mcs199 23002269 PMC3478057

[B54] Keller B. Thomson J. D. Conti E. Kudo G. (2014). Heterostyly promotes disassortative pollination and reduces sexual interference in Darwin’s primroses: evidence from experimental studies. *Funct. Ecol.* 28 1413–1425. 10.1111/1365-2435.12274

[B55] Lawton J. H. (1993). Range, population abundance and conservation. *Trends Ecol. Evol.* 8 403–419. 10.1016/0169-5347(93)90043-O 21236213

[B56] Liu S. J. Wu L. Y. Huang S. Q. (2015) Shortened anther-stigma distance reduces compatible pollination in two distylous *Primula* species. *J. Plant Ecol.* 9 224–232. 10.1093/jpe/rtv049

[B57] Lloyd D. G. (1992). Self- and cross-fertilization in plants. II. The selection of self-fertilization. *Int. J. Plant Sci.* 153 370–380. 10.1086/297041

[B58] Lloyd D. G. Webb C. J. (1992a). “The evolution of heterostyly,” in *Evolution and Function of Heterostyly*, ed. Barrett S. C. H. (Berlin: Springer-Verlag), 151–178.

[B59] Lloyd D. G. Webb C. J. (1992b). “The selection of heterostyly,” in *Evolution and Function of Heterostyly*, ed. Barrett S. C. H. (Berlin: Springer-Verlag), 179–207.

[B60] Marshall D. L. Avritt J. J. Maliakal W. Medeiros J. S. Shaner M. G. M. (2010). The impact of plant and flower age on mating patterns. *Ann. Bot.* 105 7–22. 10.1093/aob/mcp260 19875519 PMC2794063

[B61] Mast A. R. Kelso S. Conti E. (2006). Are any primroses (*Primula*) primitively monomorphic? *New Phytol.* 171 605–616. 10.1111/j.1469-8137.2006.01700.x 16866962

[B62] Moeller D. A. (2006). Geographic structure of pollinator communities, reproductive assurance, and the evolution of self-pollination. *Ecology* 87 1510–1522. 16869427 10.1890/0012-9658(2006)87[1510:gsopcr]2.0.co;2

[B63] Nishihiro J. Washitani I. Thomson J. (2000) Patterns and consequences of stigma height variation in a natural population of a distylous plant, *Primula sieboldii*. *Funct. Ecol.* 14 502–512. 10.1046/j.1365-2435.2000.00449.x

[B64] Obeso J. R. (2002). The costs of reproduction in plants. *New Phytol.* 155 321–348. 10.1046/j.1469-8137.2002.00477.x 33873312

[B65] Orsenigo S. Mondoni A. Rossi G. Abeli T. (2014). Some like it hot and some like it cold, but not too much: plant responses to climate extremes. *Plant Ecol.* 7 677–688. 10.1007/s11258-014-0363-6

[B66] Paudel B. R. Shrestha M. Burd M. Adhikari S. Sun Y.-S. Li Q. J. (2016). Coevolutionary elaboration of pollination-related traits in an alpine ginger (*Roscoea purpurea*) and a tabanid fly in the Nepalese Himalayas. *New Phytol.* 211 1402–1411. 10.1111/nph.13974 27112321

[B67] Paudel B. R. Shrestha M. Dyer A. G. Zhu X. F. Abdusalam A. Li Q. J. (2015). Out of Africa: evidence of the obligate mutualism between long Corolla tubed plant and long-tongued fly in the Himalayas. *Ecol. Evol.* 5 5240–5251. 10.1002/ece3.1784 30151127 PMC6102519

[B68] Piper J. G. Charlesworth B. Charlesworth D. (1986). Breeding system evolution in Primula vulgaris and the role of reproductive assurance. *Heredity* 56 207–217. 10.1038/hdy.1986.33

[B69] Qu R. Li X. Luo Y. Dong M. Xu H. Chen X. (2007). Wind-dragged corolla enhances self-pollination: a new mechanism of delayed self-pollination. *Ann. Bot.* 100 1155–1164. 10.1093/aob/mcm209 17881336 PMC2759251

[B70] Richards J. (2003). *Primula.* Portland, OR: Timber Press.

[B71] Richards J. H. Koptur S. (1993). Floral variation and distyly in *Guettarda scabra* (Rubiaceae). *Am. J. Bot.* 80 31–40. 10.2307/2445117

[B72] Riveros G. M. Barría O. R. Humaña P. A. M. (1995). Self-compatibility in distylous *Hedyotis salzmannii* (Rubiaceae). *Plant Syst. Evol.* 194 1–8. 10.1007/BF00983212

[B73] Rueden C. T. Schindelin J. Hiner M. C. DeZonia B. E. Walter A. E. Arena E. T. (2017). ImageJ2: ImageJ for the next generation of scientific image data. *BMC Bioinformatics* 18:529. 10.1186/s12859-017-1934-z 29187165 PMC5708080

[B74] Sagarin R. D. Gaines S. D. (2002). The ‘abundant centre’ distribution: to what extent is it a biogeographical rule? *Ecol. Lett.* 5 137–147. 10.1046/j.1461-0248.2002.00297.x

[B75] Sampson D. A. Krebs R. A. (2012). Quantitative evaluation of reciprocal herkogamy in the distylous species, *Hedyotis caerulea* (Rubiaceae). *Plant Syst. Evol.* 298 1361–1370. 10.1007/s00606-012-0642-4

[B76] Sánchez J. M. Ferrero V. Navarro L. (2013). Quantifying reciprocity in distylous and tristylous plant populations. *Plant Biol.* 15 616–620. 10.1111/j.1438-8677.2012.00720.x 23696971

[B77] Sánchez J. M. Victoria F. Luis N. (2008). A new approach to the quantification of degree of reciprocity in distylous (Sensu lato) plant populations. *Ann. Bot.* 102 463–472. 10.1093/aob/mcn111 18621965 PMC2701802

[B78] Schoen D. J. Brown A. H. D. (1991). Whole- and part-flower self-pollination in *Glycine clandestina* and G. *argyrea* and the evolution of autogamy. *Evolution* 45 1651–1664. 10.2307/2409786 28564133

[B79] Sexton J. P. McIntyre P. J. Angert A. L. Rice K. J. (2009). Evolution and ecology of species range limits. *Annu. Rev. Ecol. Evol. Syst.* 40 415–436. 10.1146/annurev.ecolsys.110308.120317

[B80] Shao J. W. Wang H. F. Fang S. P. Conti E. Zhu H. M. (2019). Intraspecific variation of self-incompatibility in the distylous plant *Primula merrilliana*. *AoB Plants* 11:lz030. 10.1093/aobpla/plz030 32489575 PMC6557196

[B81] Shao J. W. Zhang W. J. Zhang X. P. (2011). Reproductive characteristics and adaptive evolution of pin and thrum flowem in endangered species, *Primula mertilliana*. *Acta Ecol. Sin.* 31 6410–6419.

[B82] Shao J. W. Wu Y. F. Kan X. Z. Liang T. J. Zhang X. P. (2012). Reappraisal of *Primula ranunculoides* (Primulaceae), an endangered species endemic to China, based on morphological, molecular genetic and reproductive characters. *Bot. J. Linn. Soc.* 169 338–349. 10.1111/j.1095-8339.2012.01228.x

[B83] Shao J. W. Zhang X. P. Zhang Z. X. Zhu G. P. (2008). Identification of effective pollinators of *Primula merrilliana* and effects of flower density and population size on pollination efficiency. *J. Syst. Evol.* 46 537–544. 10.3724/SP.J.1002.2008.07142

[B84] Stephenson A. G. (1981). Flower and fruit abortion: proximate causes and ultimate functions. *Annu. Rev. Ecol. Syst.* 12 253–279. 10.1146/annurev.es.12.110181.001345

[B85] Takebayashi N. Morrell P. L. (2001). Is self-fertilization an evolutionary dead end? Revisiting an old hypothesis with genetic theories and a macroevolutionary approach. *Am. J. Bot.* 88 1143–1150. 10.2307/3558325 11454614

[B86] Villellas J. Ehrlén J. Olesen J. Braza R. García M. (2013). Plant performance in central and northern peripheral populations of the widespread *Plantago coronopus*. *Ecography* 36 136–145. 10.1111/j.1600-0587.2012.07425.x

[B87] Wang X. J. Barrett S. C. H. Zhong L. Wu Z. K. Li D. Z. Wang H. (2020). The genomic selfing syndrome accompanies the evolutionary breakdown of heterostyly. *Mol. Biol. Evol.* 38 168–180. 10.1093/molbev/msaa199 32761213 PMC7782863

[B88] Webb C. J. Lloyd D. G. (1986). The avoidance of interference between the presentation of pollen and stigmas in angiosperms II. Herkogamy. *N. Z. J. Bot.* 24 163–178. 10.1080/0028825X.1986.10409726

[B89] Wedderburn F. Richards A. J. (1990). Variation in within-morph incompatibility inhibition sites in heteromorphic *Primula* L. *New Phytol.* 116 149–162. 10.2307/2560424

[B90] Wu Y. Li Q. J. (2017). Phenotypic selection on flowering phenology and pollination efficiency traits between *Primula* populations with different pollinator assemblages. *Ecol. Evol.* 7 7599–7608. 10.1002/ece3.3258 29043017 PMC5632619

[B91] Yuan S. Barrett S. C. H. Duan T. Qian X. Shi M. Zhang D. (2017). Ecological correlates and genetic consequences of evolutionary transitions from distyly to homostyly. *Ann. Bot.* 120 775–789. 10.1093/aob/mcx098 28961784 PMC5691548

[B92] Zhong L. Barrett S. C. H. Wang X. J. Wu Z. K. Sun H. Y. Li D. Z. (2019). Phylogenomic analysis reveals multiple evolutionary origins of selfing from outcrossing in a lineage of heterostylous plants. *New Phytol.* 224 1290–1303. 10.1111/nph.15905 31077611

[B93] Zhou W. Barrett S. C. H. Li H. D. Wu Z. K. Wang X. J. Wang H. (2017). Phylogeographic insights on the evolutionary breakdown of heterostyly. *New Phytol.* 214 1368–1380. 10.1111/nph.14453 28176339

[B94] Zhou W. Barrett S. C. H. Wang H. Li D. Z. (2015). Reciprocal herkogamy promotes disassortative mating in a distylous species with intramorph compatibility. *New Phytol.* 206 1503–1512. 10.1111/nph.13326 25664897

[B95] Zhu X. F. Li Y. Wu G. L. Fang Z. D. Li Q. J. Liu J. Q. (2009). Molecular and morphological evidence for natural hybridization between *Primula secundiflora* franchet and P. *poissonii* franchet (Primulaceae). *Acta Biol. Crac. Ser. Bot.* 51 29–36. 10.1186/1471-2229-9-1 19123941 PMC2630931

