# Supplementary material for: Evolution of Autonomous Selfing in Marginal Habitats: Spatiotemporal Variation in the Floral Traits of the Distylous Primula wannanensis
Source: Front Plant Sci. 2021 Dec 16;12:781281. doi: 10.3389/fpls.2021.781281 (PMC8716950; doi:10.3389/fpls.2021.781281)
Supplement: Supplementary file 1 [file Data_Sheet_1.docx]

Supplementary Material

# Supplementary Figures and Tables

## Supplementary Figures


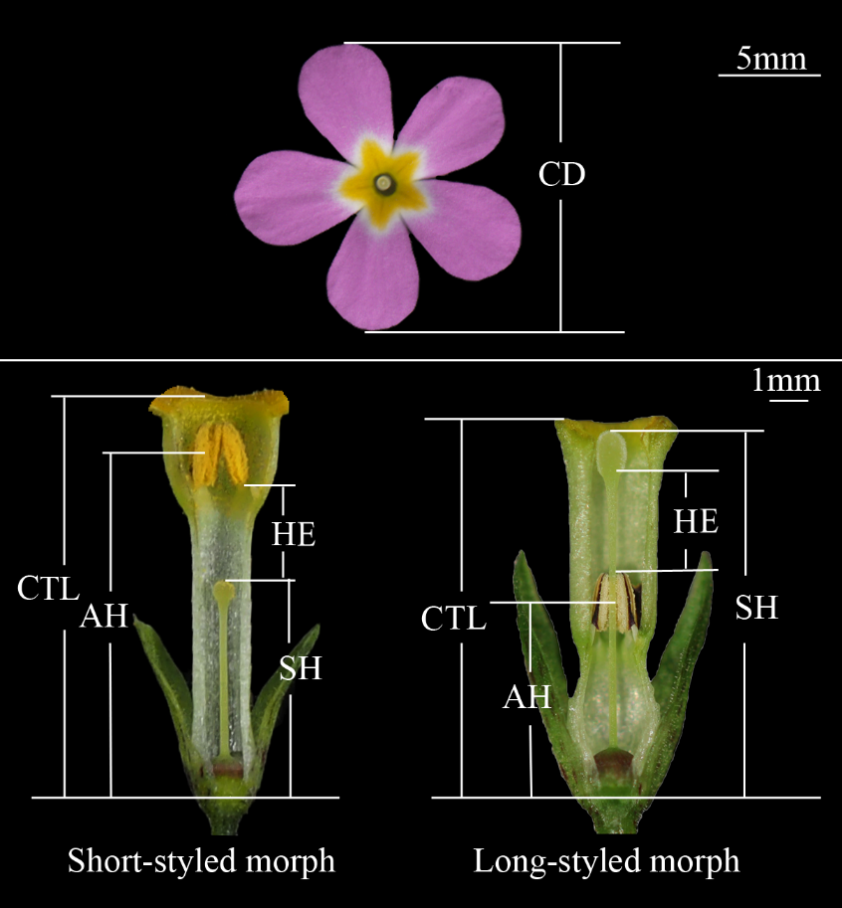


**Supplementary Figure 1.** Morphological floral traits measured in the two floral morphs, short-styled morph (S-morph) and long-styled morph (L-morph), of *Primula wannanensis*. SH, stigma height; AH, anther height; HE, herkogamy; CTL, corolla tube length; CD, corolla diameter.


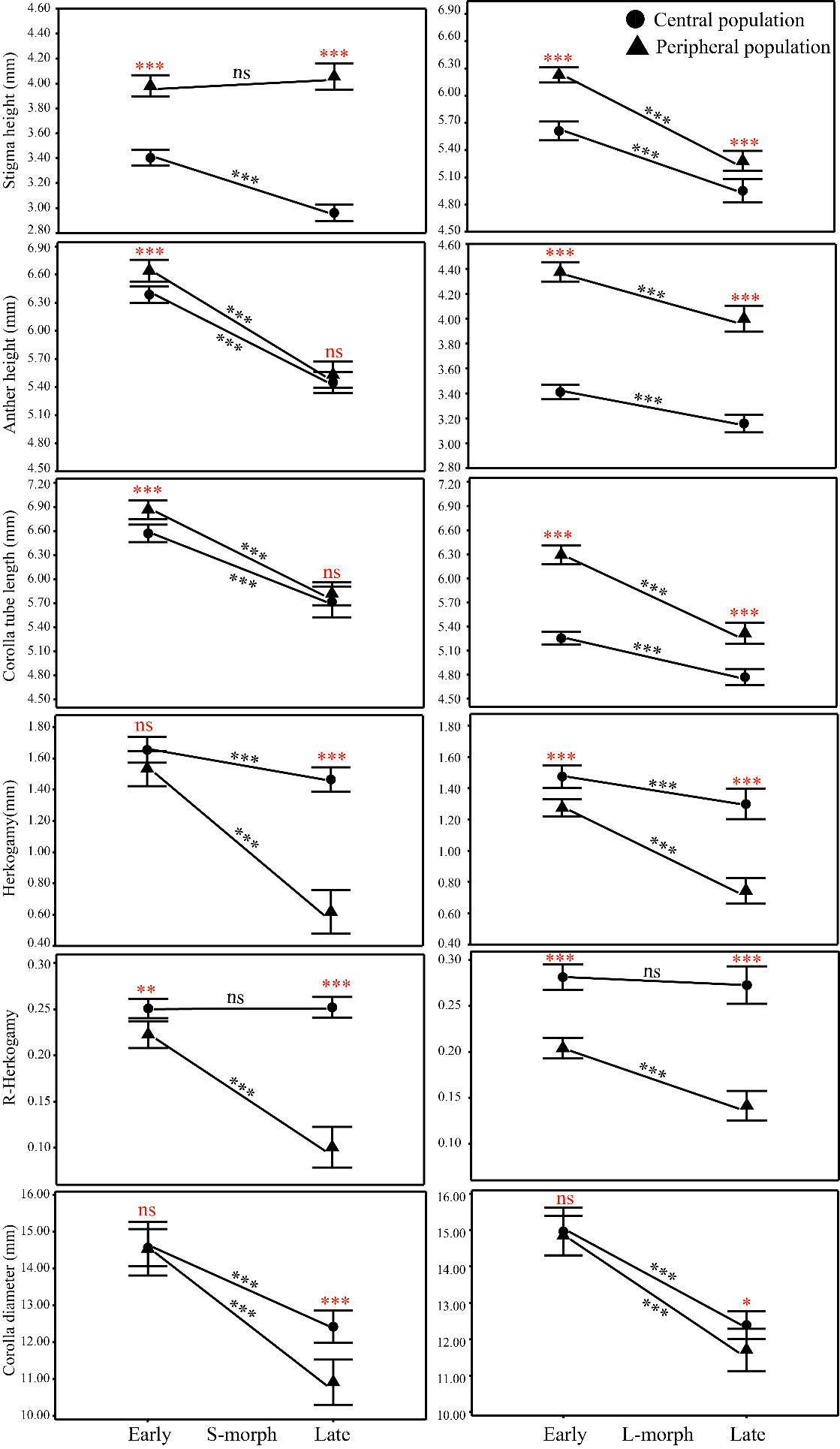


**Supplementary Figure 2.** Spatiotemporal differences in floral traits of S- and L-morphs of *Primula wannanensis* between different sites (central vs. peripheral) and between different flowering phases (early vs. late) in 2018. Data represent mean ± standard error (SE). Red asterisks (_*_) indicate significant differences between the two sites (central vs. peripheral), and black asterisks (_*_) indicate significant differences between the two time points (early vs. late).

## Supplementary Tables

**Supplementary Table 1.** Details of the *Primula wannanensis* populations used in this study.

| Site | Population  code | Location | Elevation  (m) | Latitude  (N) | Longitude  (E) | Morph frequency | population size |
| --- | --- | --- | --- | --- | --- | --- | --- |
|  |  |  |  |  |  | S : L |  |
| Centre | HDY | HuangShan | 400-700 | 118.24 | 30.18 | 0.52 : 0.48 | approx.5000 |
|  | GCN | HuangShan | 450-750 | 117.93 | 30.10 | 0.49 : 0.51 | approx.2000 |
| Periphery | YLD | ChiZhou | 200-350 | 117.84 | 30.32 | 0.53 : 0.47 | approx.1000 |
|  | QDX | ChiZhou | 250-350 | 117.78 | 30.26 | 0.54 : 0.46 | approx.800 |

**Supplementary Table 2.** Principal component analysis (PCA) of six floral traits of L- and S-morphs of *P. wannanensis* in the central and peripheral populations at different florescence stages.

| Traits | Early | | | | Late | | | |
| --- | --- | --- | --- | --- | --- | --- | --- | --- |
|  | S-morph | | L-morph | | S-morph | | L-morph | |
|  | PC1 | PC2 | PC1 | PC2 | PC1 | PC2 | PC1 | PC2 |
| Stigma height | 0.01 | 0.95 | 0.13 | 0.94 | -0.80 | 0.51 | 0.19 | 0.83 |
| Anther height | 0.80 | 0.50 | 0.91 | 0.29 | 0.45 | 0.73 | -0.83 | 0.42 |
| Corolla tube length | 0.81 | 0.54 | 0.79 | 0.51 | 0.27 | 0.87 | -0.52 | 0.77 |
| Herkogamy | 0.89 | -0.43 | -0.80 | 0.55 | 0.97 | -0.08 | 0.93 | 0.25 |
| R-Herkogamy | 0.67 | -0.71 | -0.93 | 0.31 | 0.88 | -0.32 | 0.97 | 0.11 |
| Corolla diameter | 0.41 | 0.04 | -0.10 | 0.64 | 0.46 | 0.42 | 0.35 | 0.72 |

Notes: Variation in individual traits explained by the first two principal components (PCs) is shown.

**Supplementary Table 3.** PCA of six floral traits of L- and S- morphs of *P. wannanensis* at the early and late florescence stages at different sites.

| Traits | Centre | | | | Periphery | | | |
| --- | --- | --- | --- | --- | --- | --- | --- | --- |
|  | S-morph | | L-morph | | S-morph | | L-morph | |
|  | PC1 | PC2 | PC1 | PC2 | PC1 | PC2 | PC1 | PC2 |
| Stigma height | 0.61 | -0.60 | 0.87 | 0.29 | -0.35 | 0.91 | 0.98 | 0.06 |
| Anther height | 0.85 | 0.16 | -0.14 | 0.81 | 0.93 | 0.24 | 0.37 | 0.88 |
| Corolla tube length | 0.90 | -0.11 | 0.33 | 0.86 | 0.94 | 0.29 | 0.76 | 0.56 |
| Herkogamy | 0.61 | 0.72 | 0.92 | -0.23 | 0.97 | -0.21 | 0.81 | -0.56 |
| R-Herkogamy | -0.02 | 0.86 | 0.79 | -0.55 | 0.94 | -0.29 | 0.67 | -0.73 |
| Corolla diameter | 0.73 | -0.13 | 0.59 | 0.38 | 0.74 | 0.41 | 0.84 | 0.15 |

**Supplementary Table 4.** Univariate analysis of variance of the effects of space (central vs. peripheral sites) and time (early vs. late florescence stages) on six floral traits of S- and L-morphs.

| Variable | S-morph | | | L-morph | | |
| --- | --- | --- | --- | --- | --- | --- |
|  | *d.f* | *F* | *P* | *d.f* | *F* | *P* |
| Stigma height |  |  |  |  |  |  |
| Space | 1 | 1077.98 | **<0.001** | 1 | 42.80 | **<0.001** |
| Time | 1 | 7.56 | **0.006** | 1 | 537.26 | **<0.001** |
| Space * Time | 1 | 64.05 | **<0.001** | 1 | 27.03 | **<0.001** |
| Anther Height |  |  |  |  |  |  |
| Space | 1 | 44.37 | **<0.001** | 1 | 628.29 | **<0.001** |
| Time | 1 | 449.69 | **<0.001** | 1 | 49.56 | **<0.001** |
| Space * Time | 1 | 48.53 | **<0.001** | 1 | 9.80 | **0.002** |
| Corolla tube length |  |  |  |  |  |  |
| Space | 1 | 54.90 | **<0.001** | 1 | 217.81 | **<0.001** |
| Time | 1 | 505.49 | **<0.001** | 1 | 411.91 | **<0.001** |
| Space * Time | 1 | 48.85 | **<0.001** | 1 | 74.69 | **<0.001** |
| Herkogamy |  |  |  |  |  |  |
| Space | 1 | 222.56 | **<0.001** | 1 | 210.43 | **<0.001** |
| Time | 1 | 339.78 | **<0.001** | 1 | 123.45 | **<0.001** |
| Space * Time | 1 | 177.90 | **<0.001** | 1 | 15.87 | **<0.001** |
| Relative herkogamy |  |  |  |  |  |  |
| Space | 1 | 386.21 | **<0.001** | 1 | 349.44 | **<0.001** |
| Time | 1 | 153.80 | **<0.001** | 1 | 43.24 | **<0.001** |
| Space * Time | 1 | 190.62 | **<0.001** | 1 | 7.90 | **0.005** |
| Corolla diameter |  |  |  |  |  |  |
| Space | 1 | 37.11 | **<0.001** | 1 | 13.35 | **<0.001** |
| Time | 1 | 324.93 | **<0.001** | 1 | 239.50 | **<0.001** |
| Space * Time | 1 | 3.84 | 0.05 | 1 | 7.64 | **0.006** |

Notes: Values in bold indicate significant *P*-values (*P* < 0.05)

**Supplementary Table 5.** Pearson correlation coefficients of floral traits of *P. wannanensis* plants.

| Floral traits | SH | AH | CTL | HE | RHE | CD |
| --- | --- | --- | --- | --- | --- | --- |
| SH | - | 0.65** | 0.70** | 0.31** | -0.03 | 0.21** |
| AH | 0.48** | - | 0.86** | -0.44** | -0.68** | -0.00 |
| CTL | 0.52** | 0.87** | - | -0.29** | -0.64** | 0.09 |
| HE | -0.36** | 0.47** | 0.48** | - | 0.90** | 0.07 |
| RHE | -0.60** | 0.18** | 0.10 | 0.91** | - | 0.04 |
| CD | 0.02 | 0.14** | 0.26** | 0.19** | 0.12* | - |

Notes: Asterisks indicate significant differences (**P* < 0.05, ***P* < 0.01). Values on the left side of the diagonal represent correlation coefficients among floral traits in the S-morph, and values on the right side of the diagonal represent correlation coefficients among floral traits in the L-morph.

**Supplementary Table 6.** Multivariate analysis of variance of the effects of site (space), flowering phase (time), and their interaction on the overall floral traits of L- and S-morphs measured in 2018.

| Morphs | Variable | Wilks’λ | *d.f* | *F* | *P* |
| --- | --- | --- | --- | --- | --- |
| S-morph | Space | 0.34 | 6,241 | 78.42 | <0.0001 |
|  | Time | 0.42 | 6,241 | 53.57 | <0.0001 |
|  | Space * Time | 0.68 | 6,241 | 18.59 | <0.0001 |
| L-morph | Space | 0.37 | 6,237 | 108.41 | <0.0001 |
|  | Time | 0.41 | 6,237 | 26.14 | <0.0001 |
|  | Space * Time | 0.83 | 6,237 | 8.25 | <0.0001 |

**Supplementary Table 7.** Univariate analysis of variance of the effects of space (central vs. peripheral sites) and time (early vs. late florescence) on six floral traits of S- and L-morphs measured in 2018.

| Variable | S-morph | | | L-morph | | |
| --- | --- | --- | --- | --- | --- | --- |
|  | *d.f* | *F* | *P* | *d.f* | *F* | *P* |
| Sigma height |  |  |  |  |  |  |
| Space | 1 | 428.46 | **<0.001** | 1 | 70.28 | **<0.001** |
| Time | 1 | 20.70 | **0.006** | 1 | 202.35 | **<0.001** |
| Space * Time | 1 | 40.82 | **<0.001** | 1 | 6.52 | **0.011** |
| Anther height |  |  |  |  |  |  |
| Space | 1 | 8.81 | **0.003** | 1 | 539.37 | **<0.001** |
| Time | 1 | 323.53 | **<0.001** | 1 | 65.35 | **<0.001** |
| Space * Time | 1 | 2.31 | 0.13 | 1 | 2.55 | 0.112 |
| Corolla tube length |  |  |  |  |  |  |
| Space | 1 | 6.97 | **0.009** | 1 | 225.00 | **<0.001** |
| Time | 1 | 220.57 | **<0.001** | 1 | 190.60 | **<0.001** |
| Space * Time | 1 | 4.74 | **0.03** | 1 | 21.84 | **<0.001** |
| Herkogamy |  |  |  |  |  |  |
| Space | 1 | 88.58 | **<0.001** | 1 | 84.08 | **<0.001** |
| Time | 1 | 115.57 | **<0.001** | 1 | 73.54 | **<0.001** |
| Space * Time | 1 | 49.99 | **<0.001** | 1 | 18.48 | **<0.001** |
| Relative herkogamy |  |  |  |  |  |  |
| Space | 1 | 149.68 | **<0.001** | 1 | 156.36 | **<0.001** |
| Time | 1 | 67.28 | **<0.001** | 1 | 18.33 | **<0.001** |
| Space * Time | 1 | 70.17 | **<0.001** | 1 | 10.49 | **0.001** |
| Corolla diameter |  |  |  |  |  |  |
| Space | 1 | 7.34 | **0.007** | 1 | 1.105 | 0.294 |
| Time | 1 | 103.26 | **<0.001** | 1 | 111.92 | **<0.001** |
| Space * Time | 1 | 6.75 | **0.01** | 1 | 2.22 | 0.137 |

Notes: Values in bold indicate significant *P* values (*P* < 0.05).
